# Physiological and Psychological Effects of Visual Stimulation with Green Plant Types

**DOI:** 10.3390/ijerph182412932

**Published:** 2021-12-08

**Authors:** Ji-Eun Jeong, Sin-Ae Park

**Affiliations:** 1 Department of Bio and Healing Convergence, Graduate School, Konkuk University, Seoul 05029, Korea; uuo9564@naver.com; 2 Department of Systems Biotechnology, Konkuk Institute of Technology, Konkuk University, Seoul 05029, Korea

**Keywords:** brain waves, electroencephalography, horticultural therapy, profile of mood states, semantic differential methods

## Abstract

This study was designed to assess the physiological and psychological benefits of visually looking at foliage plants in adults. This study involved 30 adults in their 20s (11 males, 19 females), and using a crossover design, participants looked at four different types of visual stimuli, namely, real plants, artificial plants, a photograph of plants, and no plants for 5 min. Brain waves were measured while viewing each type of plant, and a subjective evaluation of emotions was performed after each visual stimulus. Semantic differential methods (SDM) and Profile of Mood States (POMS) were used for the subjective evaluation. During the real plant visual stimulation, relative theta (RT) power spectrum was increased in the bilateral occipital lobes, while relative high beta (RHB) power spectrum was reduced in the left occipital lobe, indicating a reduction in stress, anxiety, and tension. The subjective survey results revealed that when looking at real plants, the participants exhibited significantly higher “comfort,” “natural,” and “relaxed” scores as well as an increase in positive mood conditions. In conclusion, among the four types of plants, visual stimulation with real plants induces physiological relaxation in adults and has a positive psychological effect.

## 1. Introduction

### 1.1. The Effects of Natural Environment on Human Psychophysiology

Modern urbanized societies are isolating people from their natural environment and reducing contact with nature [1]. Environmental stress (e.g., neon signs, noise, and air pollution) exacerbates psychological health by significantly increasing fatigue and stress in people living in cities [2]. Nature, on the other hand, provides social and psychological benefits, improves human quality of life, promotes positive feeling, and accelerates physiological recovery [3,4]. As such, the quality of urban environments contributes to human health and well-being, and the incorporation of nature into urban environments helps individuals cope with negative moods and stress [5,6]. In fact, Cohen [7] argues that modern people prefer the natural environment to that of urban ones as an escape from the overload of modern society, and that psychological recovery can be achieved through exposure to the natural environment. Additionally, the Savannah theory [8] further supports this idea, suggesting that humans instinctively prefer nature.

Kaplan and Kaplan [9] argued that the inherent eco-environment elements in nature induce people’s attention, which effectively restores attention as well as positively affects mental fatigue and relives stress. In line with this, one study reported that when humans are exposed to external stress, their emotional, attentive, and physical recovery is faster and more complete when exposed to a natural environment compared to an urban environment [2]. Based on these theories, numerous studies comparing the benefits of urban versus natural environments have been conducted. For example, decreased cortisol concentrations, reduced pulse, increased stability of the autonomic nervous system [10], decreased blood pressure, and increased alpha and theta waves [11] have all been reported in individuals exposed to a natural environment. It has also been reported that the proportion of green spaces in living environments affects general health, in addition to proving significant social and emotional benefits [6,12].

### 1.2. A Prior Study on the Effects of Plants on Human

In addition, plant-based activities are known to provide significant benefits to individuals. For example, horticultural activities in children have been shown to improve peer relationships, social skills, self-efficacy, and emotional intelligence as well as reduce the incidence of depression [13,14,15,16]. Furthermore, horticulture activities been shown to reduce depression and anxiety, increase self-identify, and increase levels of brain-derived neurotrophic factor (BDNF) in older adults, leading to improved cognitive function [17,18,19]. Horticultural activities can also help improve hand function and improve muscle activity in the upper and lower extremities [20,21,22,23].

Various studies have been conducted assessing the physiological and psychological changes that occur in the visual stimulation with green plants in adults. For example, one study comparing white walls to plants as visual stimuli, found that looking at plants increased the participant’s alpha waves and blood pressure while decreasing their heart rate [24]. Furthermore, another study comparing no plants to real plants as visual stimuli found that when the participants looked at real plants, there was a reduction in hemoglobin oxide levels in the prefrontal lobe and they reported feeling physiologically relaxed [25]. In addition, plants are known to provide a sense of comfort, and the feeling of being in nature also positively impacts creativity and concentration [26,27,28]. Another study found that when subjects looked at the natural environment indoors, they experienced a reduction in anxiety and tension, increased alpha and beta waves, and a reduction in blood pressure [29,30].

Taken together, the importance of the natural environment and its positive effects on humans are confirmed. Although there are various of psychophysiological studies assessing exposure to natural environments in adults, studies assessing the effects of different types of exposure to green plants are lacking. Therefore, this study was conducted to measure changes in brain waves and subjective emotional changes induced by various type of green plant visual stimuli in adults.

## 2. Materials and Methods

### 2.1. Participants

This study included 11 males and 19 females in their 20s. This study referred to previous studies on psychophysiology and set the number of subjects to 30. Previous studies on the psycho-physiology of horticultural activities were conducted with 30 subjects in a single group without a control group [27,31]. To recruit participants, other subjects were introduced and recruited through subjects who completed the study using the snowball sampling method. Only right-handed participants were included in this study based on previous research demonstrating that people of different handedness differed in their brain activity by Tarkka and Hallett [32]. In addition, it was based on those who are not currently suffering from a specific disease [24]. Participants were asked to fast for 2 h before the experiment in order to eliminate the potential effects of caffeine that naturally occurs in various foods, which may stimulate the brain [33]. Before the experiment, participants received an explanation of the details of the study, after which they provided their informed consent, and their demographic information was collected through a questionnaire. Subsequently, the participant’s height, weight, and body mass index (BMI) (ioi 353; Jawon Medical, Gyeongsan, South Korea) were measured. This study was conducted with the approval of Konkuk University’s Institutional Review Board (7001355–202004-HR-376).

### 2.2. Experimental Environment

**Figure 1:**
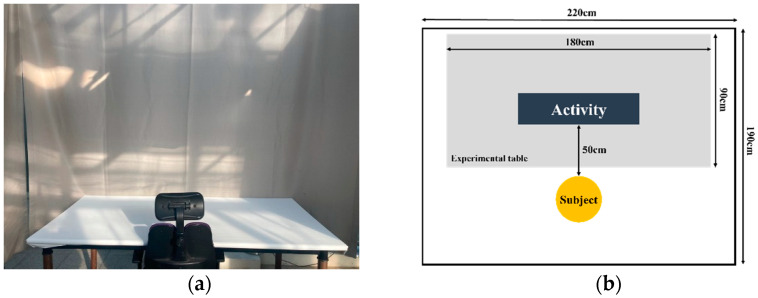
Experimental condition: (**a**) experimental room; (**b**) arrangement during the experiment.

This study was conducted in a space (220 cm × 190 cm) at Konkuk University in Seoul, South Korea. The conditions of the experimental space were as follows: 25.22 ± 2.79 °C; average relative humidity, 27.16 ± 9.87%, and average light intensity, 3577.90 ± 1968.60 lux. During the experiment, an ivory curtain was placed on the front and both sides of the experimental space to minimize external stimulation, and white sheets were attached to the desk. The height of the chair was adjusted according to the height of the participants, and the chair was placed in the center of the desk (Figure 1).

### 2.3. Experimental Materials and Treatment

**Figure 2:**
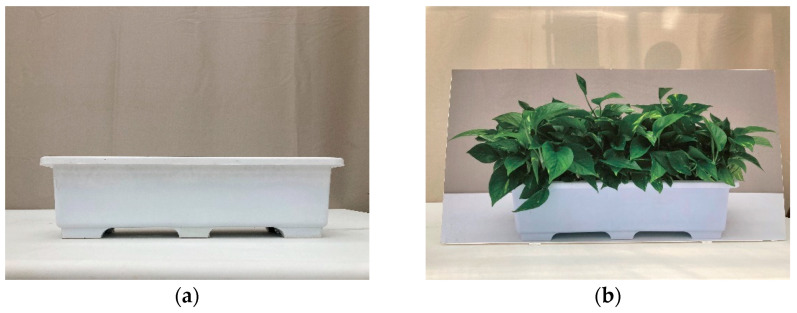

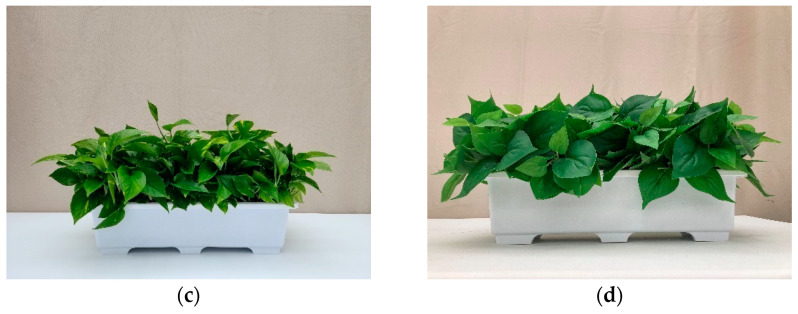
Experimental materials and set-ups used as the visual stimuli: (**a**) no plants; (**b**) photograph of plants; (**c**) real plants; (**d**) artificial plants.

In this study, visual stimuli were used with four different plant types (Figure 2): (1) no plants, (2) a photograph of plants, (3) real plants, and (4) artificial plants. White flowerpots (width 45 cm, height 15 cm) were used for all treatments. Each treatment was prepared as follows: (1) no plants: Horticultural soils were used without planting plants; (2) a photograph of plants: A life-size color photograph of the living plants that we used as real plant stimulants was printed; (3) real plants: 13 Pots of *Epipremnumaureum* were planted in a flowerpot and arranged to look full of green; (4) artificial plants: artificial plants similar to the *E. aureum* were used as real plant stimulants.

### 2.4. Experimental Procedure

**Figure 3:**
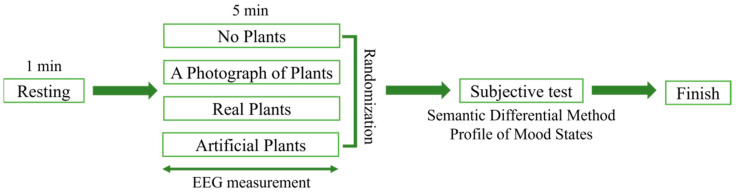
Experimental protocol. EEG, electroencephalography.

Before visual stimulus began, the participant was directed to look at the white screen for 1 min in order to minimize visual stimulation. Visual stimulation processing of the four different stimuli was randomized, and brain waves measured for 5 min per treatment (Figure 3). 

**Figure 4:**
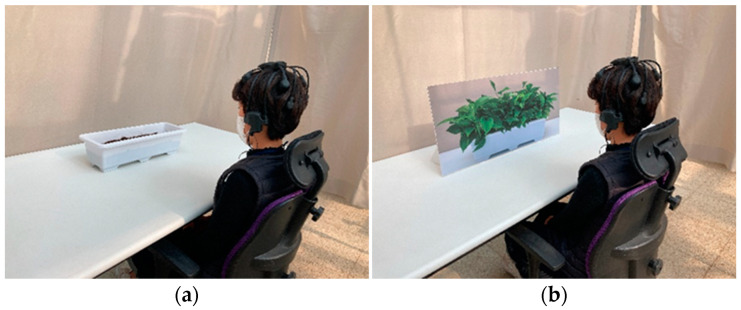

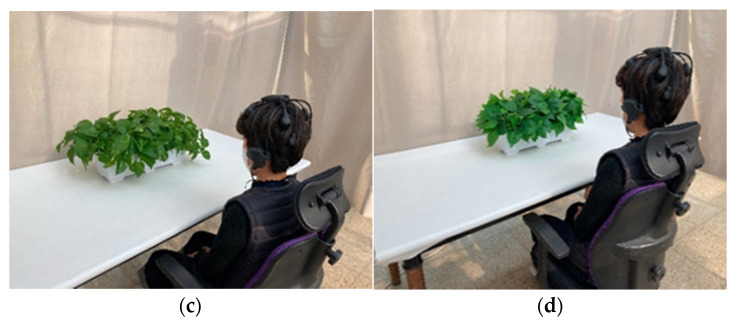
Experimental viewing conditions: (**a**) no plants; (**b**) a photograph of plants; (**c**) real plants; (**d**) artificial plants.

While measuring brain waves, the participants were directed to not move or speak. After the visual stimulation of each treatment, two questionnaires were conducted to record the subjective emotions of the participants, followed by 1 min of rest, before moving on to the next visual stimulation. The experiment concluded after visual stimulation of all four treatments was completed. The average experimental time per subject was approximately 29.68 ± 4.71 min (Figure 4).

### 2.5. Measurement

A wireless dry electroencephalography (EEG) device (Quick-20l Cognionics, San Diego, CA, USA) was used to measure the participants’ brain waves during visual stimulation of each treatment. This EEG device is a dry electrode system rather than a wet device with electrolyte gel, which minimizes the risk of electric shock. In addition, it has the advantage of quicker setup time, increased versatility, and improved mobility [34]. This device has been safety certified by the European Commission and the Federal Communications Commission [31]. Brain waves were measured using a brain mapping program (Bioteck Analysis Software; Daejeon, Korea) to determine the average EEG during the experiment.

**Figure 5:**
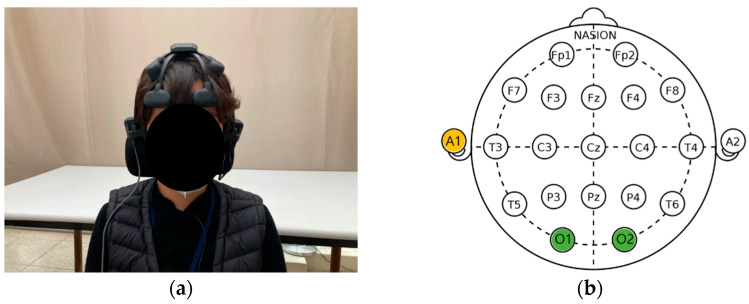
Wearing instrument: (**a**) wireless dry electroencephalography device (Quick-20; Cognionics, San Diego, CA, USA); (**b**) International electrode arrangement [35].

The electrodes were attached to the left earlobe (A1), according to the International 10–20 Electrode Placement System [35]. In addition, electrodes were attached to a total of eight channels to measure brain waves: the left prefrontal lobe (Fp1), right prefrontal lobe (Fp2), left frontal lobe (F3), right frontal lobe (F4), left parietal lobe (P3), right parietal lobe (P4), left occipital lobe (O1), and right occipital lobe (O2) (Figure 5). The reference electrode was attached to the left earlobe (A1) and the EEG was measured using the O1 and O2 channels, which are involved in vision [36].

The Semantic differential method (SDM) developed by Osgood [37] and measures emotions with adjectives. SDM is 13-point Likert scale consisting of three items: “comfortable-uncomfortable,” “natural-artificial,” and “relaxed-awaken.” Higher scores indicate a positive emotional state. Profile of mood states (POMS) were developed by McNair et al. [38]. POMS measure a transient mood or emotional state that varies with the environment in which the subject is present. The questionnaire contains 30 questions, consisting of tension-anxiety (T-A), depression (D), anger-hostility (A-H), fatigue (F), confusion (C), and vigor (B). The total mood disorder (TMD) score is evaluated by summing the values of each question [(T-A) + (D) + (A-H) + (F) + (C)-(V)]. Lower TMD values indicate a positive emotional state.

### 2.6. Data Analysis

The EEG data were analyzed using Biotech Analysis Software (Daejeon, South Korea). Brain waves from the cerebral cortex were classified as theta (4–8 Hz), alpha (8–13 Hz), beta (13–30 Hz), and gamma (30–50 Hz) based on frequency [39]. Each frequency can be interpreted in a different sense: theta waves indicate shallow sleep, alpha waves indicate relaxation, beta indicate mental activity, and gamma waves indicate anxiety or excitement [40]. In this study, relative theta (RT) power spectrum, meaning relaxation, and relative high beta (RHB) power spectrum, meaning stress, were analyzed (Table 1).

The results of the EEG, SDM, and POMS for each stimulus were analyzed using IBM SPSS Statistics for Windows (version25; IBM Corp., Armonk, NY, USA). One-way analysis of variance, and Duncan’s multiple range tests were performed. All significance levels were set to *p* < 0.05. To analyze demographic information, Microsoft excel (Microsoft Office 365 ProPlus; Microsoft, Redmond, WA, USA) was used to determine the descriptive statistics.

## 3. Results

### 3.1. Descriptive Characteristics

The average age of the subjects in this study was 26.4 ± 1.9 years, with 11 (36.7%) men and 19 (63.3%) women for a total of 30 participants. The average height was 165.6 ± 9.9 cm, and the average weight was 64.2 ± 12.5 kg. The average BMI was 23.4 ± 3.3 kg∙m^−2^, which is within the normal range according to World Health Organization standards of the Korea Centers for Disease Control and Prevention (Table 2).

### 3.2. Electroencephalography (EEG)

The RT power spectrum was significantly higher in the left occipital lobe when participants were looking at the real plant (95% CI 0.18–0.22), and the RT power spectrum was significantly higher in the right occipital lobe when participants were looking at either the real (95% CI 0.18–0.21) or artificial plants (95% CI 0.18–0.21) (*p* < 0.05). The RHB power spectrum was significantly lower in the left occipital lobe when participants were looking at the real plant (95% CI 0.17–0.18) (*p* < 0.05). However, no significant difference was found in the right occipital lobe (Table 3).

Increased RT power spectrum is indicative of physiological relaxation [41,42], and increased RHB power spectrum indicates increased stress and anxiety [43,44]. Therefore, in this study, when looking at a real plants, the participants experienced physiological relaxation as well as a reduction in stress and anxiety.

### 3.3. Results of the Subjective Mood Evaluation According to the Type of Visual Stimulation Presented

**Figure 6:**
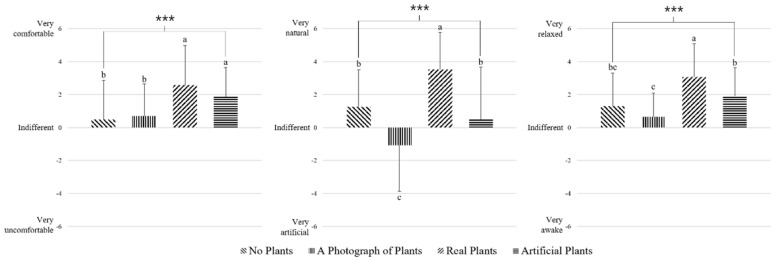
Comparisons of a semantic differential method (SDM) for each visual stimulation. *** *p* < 0.001 according to the one-way analysis of variance. Post hoc analysis: a > b > c according to Duncan’s multiple range tests.

An evaluation of SDM based on visual stimulation of green plants showed significantly higher “comfort” (*p* < 0.001) when looking at real plants and artificial plants, and significantly higher “natural” (*p* < 0.001) and “relaxed” (*p* < 0.001) scores when looking at real plants (Figure 6).

**Figure 7:**
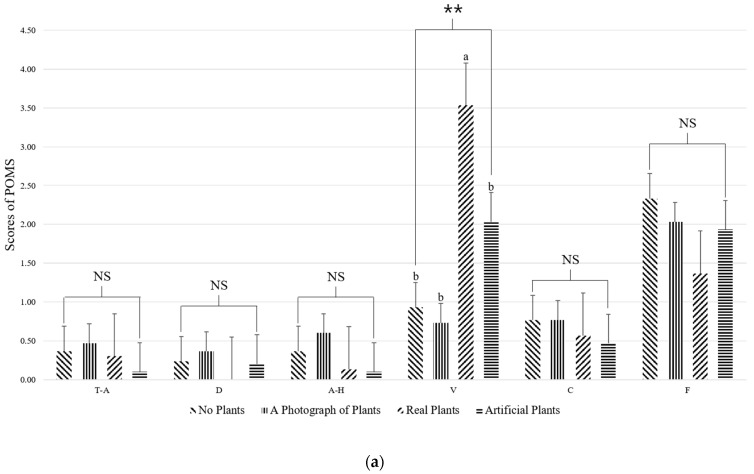

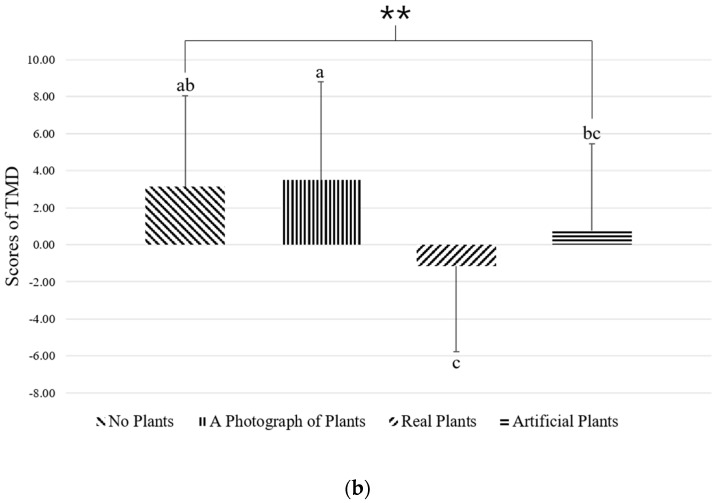
(**a**) Comparisons of tension-anxiety (T-A), depression-dejection (D), anger-hostility (A-H), vigor (V), confusion (C), and fatigue (F) based on the Profile of Mood States (POMS) questionnaire for each activity; (**b**) Comparisons of the total mood disturbance (TMD) score in the POMS questionnaire between conditions. NS = non-significant, ** *p* < 0.01 according to a one-way analysis of variance. Post hoc analysis: a > b > c according to Duncan’s multiple range tests.

The POMS was divided into six areas for analysis. The vigor (V) score was significantly higher when looking at real plants compared to the other treatments (*p* < 0.01) (Figure 7a). Participants also showed significantly lower TMD values when looking at real plants (*p* < 0.01) (Figure 7b).

## 4. Discussion

This study aimed to identify brainwave changes caused by four different types of green plant visual stimuli in adults. We found that RT power spectrum was significantly increased in the occipital lobe and that the RHB power spectrum was significantly decreased when the participants looked at real plants (Table 3). In other words, looking at real plants can induce physiological relaxation and reduce stress, tension, and anxiety.

The occipital lobe measured in this study constitutes the posterior part of the cerebral hemisphere [45], which is an important area of the central nervous system responsible for vision as well as integrating visual information [36,46]. Importantly, theta waves occur at a frequency of 4–8 Hz [40] and appear when a person is in shallow sleep with their eyes closed or in a state of deep relaxation, such as during meditation or hypnosis [41,42]. It is also associated with deep internalization and quiet physical, emotional, and critical thinking activities [47]. The beta wave improves concentration and attention with 13 to 30 Hz of fast waves [40]. Beta waves are subdivided into low beta waves, medium beta waves, and high beta waves, with high beta waves ranging from 20 to 30 Hz [48], appearing in muscle tension and elevated blood pressure, tension and anxiety, and stress [43,44]. Therefore, in this study, the RT power spectrum increases and the RHB power spectrum decreases, producing a feeling of comfort simply by looking at a real plant, which leads to a clear mind as well as a reduction in anxiety and stress.

The results of the SDM revealed that while participants felt comfortable when looking at real or artificial plants, they felt natural and relaxed when looking at real plants (Figure 6). Based on the results from the POMS questionnaire, when looking at real plants, participants felt vigor and their mood was improved (Figure 7). Importantly, plants have been previously reported to positively influence mood and improve the emotional state of humans. For example, Elsadek et al. [49] found that when looking at a green wall with plants versus a normal wall without plants, the participants felt comfortable, a sense of nature, relaxed, and had reduced TMD scores. Furthermore, when looking at real plants or no plants, participants felt comfortable, a sense of nature, and were relaxed, while when looking at real plants, their vigor score was high, and the TMD score was low [25]. Finally, visual stimulation studies comparing real plants and no plants have found lower blood pressure and lower anxiety scores in participants looking at real plants [50]. Along these lines, we found that the subjective emotions and mood states were positive when participants were looking at the real plants, which is thought to have influenced the subjective state of the physiological relaxation resulting from the increase in RT power spectrum and the decrease in RHB power spectrum measured via EEG.

The color green is linked to positive emotions such as nature, comfort, and peace [51,52] and is characterized by low anxiety, comfort, and stability [53]. Furthermore, green increases creativity compared to other colors [54], and reduces fatigue and anxiety as well as producing a positive psychological response characterized by high vitality and relaxation [55]. In fact, a previous study found participant’s preferred green plants over plants of other colors, and that looking at green plants reduced prefrontal cortex cerebral blood flow, which was effective in promoting positive reactions such as relaxation and vitality [56]. Wilson’s [57] Biophilia theory argues that humans have a positive inheritance to nature, preferring the natural environment, and developing emotional bonds with other living creatures. Furthermore, Kaplan and Kaplan [9] proposed the attention restoration theory, arguing that exposure to the natural environment is effective in restoring one’s attention. These theories related to the natural environment are supported by the fact that humans are attracted to green plants, and that green visual stimuli induces a state of physiological relaxation and reduces stress.

A previous study related to visual stimulation of plants found that brain functions were more active when participants were exposed to green plants [58]. Studies assessing changes in the autonomic nervous system after looking at a green landscape compared to an urban landscape found increased theta waves [11] and an increase in parasympathetic nerve activity [49]. In addition, a study comparing a real plant to no plant as visual stimuli found significantly lower levels of oxidized hemoglobin in the right prefrontal lobe when the participants were looking at the real plant, indicating a physiologically relaxed state [25]. Another study that compared the visual stimuli of living flowers and artificial flowers found that sympathetic nerve activity was decreased when participants were looking at the living flowers [26]. While previous studies compared visual stimuli according to the presence or absence of plants, this study presented different types of green plant visual stimuli. We found that real plants provide comfort to humans by inducing a state of relaxation and reducing stress. Recently, indoor plants have been attracting attention due to the effects of air purification and emotional aspects, and based on the results of this study, placing real plants indoors rather than artificial plants or plant photographs may be more effective in terms of psychophysiology and psychology. The limitations of this study are that it was not possible to investigate various age groups due to the age limitation, as the subjects were adults in their 20s, and because the subjects were artificially selected using the snowball sampling method. Therefore, it is thought that it will be difficult to apply the results of this study to various age groups. In the future, it is necessary to apply it to various age groups, as it is necessary to randomly recruit subjects and conduct experiments. In addition, further research will be needed in consideration of the color, shape, and texture of plants.

## 5. Conclusions

This study is a study to measure the psychophysiology and psychological response of adults by categorizing green plants. As a result, visual stimulation from real plants was effective in inducing physiological and psychological relaxation in adults. It is thought that continuous exposure of green plants will help improve human quality of life by eliciting positive emotions. This study demonstrated why humans should be closer to plants, and based on the results of this study, it could be used to apply and develop programs using green plants.

## Figures and Tables

**Table 1:** EEG power spectrum indicators used in the study [39].

Analysis Indicators	The Full Name of the EEG Power Spectrum Indicator	Wavelength Range (Hz)
RT	Relative theta power spectrum	(4–8)/(4–50)
RHB	Relative high beta power spectrum	(20–30)/(4–50)

**Table 2:** Descriptive characteristics (n = 30).

Variable	Male (n = 11)	Female (n = 19)	Total (N = 30)
	Mean ± SD ^1^		
Age (years)	26.36 ± 1.96	26.47 ± 1.90	26.43 ± 1.89
Height (cm)	175.55 ± 3.14	159.56 ± 7.21	165.62 ± 9.87
Body weight (kg)	75.28 ± 9.34	57.34 ± 8.74	64.15 ± 12.49
Body mass index (kg∙m^−2^) ^2^	23.43 ± 3.26	22.83 ± 3.43	23.43 ± 3.26

^1^ Standard deviation ^2^ Body mass index = Weight/Height^2^.

**Table 3:** Results of the relative theta (RT) and relative high beta (RHB) power spectrum according to electroencephalography (EEG).

EEG	Activity	RT ^1^		RHB ^2^	
		O1 ^3^	O2 ^4^	O1	O2
		Mean ± SD ^5^			
Total (N = 30)	No plants	0.16 ± 0.04 b ^7^	0.17 ± 0.04 b	0.19 ± 0.02 a	0.19 ± 0.02
	A photograph of plants	0.18 ± 0.04 ab	0.18 ± 0.03 ab	0.19 ± 0.02 a	0.18 ± 0.02
	Real plants	0.20 ± 0.06 a	0.19 ± 0.04 a	0.17 ± 0.02 b	0.18 ± 0.02
	Artificial plants	0.19 ± 0.04 ab	0.19 ± 0.04 a	0.18 ± 0.02 ab	0.18 ± 0.02
	P ^6^	0.015 *	0.017 *	0.015 *	0.226 ^NS^

^1^ RT power spectrum was calculated by (theta (4 to 8 Hz) power)/(total frequency (4 to 50 Hz) power). ^2^ RHB power spectrum was calculated by (high beta (20 to 30 Hz) power)/(total frequency (4 to 50 Hz) power). ^3^ O1 = left occipital lobe ^4^ O2 = right occipital lobe. ^5^ Standard deviation. ^6^ Statistical significance as determined using one-way analysis of variance. NS = Nonsignificant, * *p* < 0.05 by one-way analysis of variance. ^7^ Post hoc analysis: a > b according to Duncan’s multiple range tests.

## Data Availability

The datasets generated for this study are available on request to the corresponding author.

## References

[1] Stilgoe J.R. (2001). Gone barefoot lately?. Am. J. Prev. Med..

[2] Ulrich R.S., Simons R.F., Losito B.D., Fiorito E., Miles M.A., Zelson M. (1991). Stress recovery during exposure to natural and urban environments. J. Environ. Psychol..

[3] Chiesura A. (2004). The role of urban parks for the sustainable city. Landsc. Urban Plan..

[4] Tyrväinen L., Ojala A., Korpela K., Lanki T., Tsunetsugu Y., Kagawa T. (2014). The influence of urban green environments on stress relief measures: A field experiment. J. Environ. Psychol..

[5] Frumkin H. (2001). Beyond toxicity: Human health and the natural environment. Am. J. Prev. Med..

[6] Maas J., Verheij R.A., Groenewegen P.P., de Vries S., Spreeuwenberg P. (2006). Green space, urbanity, and health: How strong is the relation?. J. Epidemiol. Community Health.

[7] Cohen S. (1978). Environmental load and the allocation of attention. Adv. Environ. Psych..

[8] Orians G.H. (1986). An ecological and evolutionary approach to landscape aesthetics. Landsc. Mean. Values.

[9] Kaplan R., Kaplan S. (1989). The Experience of Nature: A Psychological Perspective.

[10] Lee J., Park B.-J., Tsunetsugu Y., Ohira T., Kagawa T., Miyazaki Y. (2011). Effect of forest bathing on physiological and psychological responses in young Japanese male subjects. Public Health.

[11] Jiang M., Hassan A., Chen Q., Liu Y. (2019). Effects of different landscape visual stimuli on psychophysiological responses in Chinese students. Indoor Built Environ..

[12] Markevych I., Tiesler C.M., Fuertes E., Romanos M., Dadvand P., Nieuwenhuijsen M.J., Berdel D., Koletzko S., Heinrich J. (2014). Access to urban green spaces and behavioural problems in children: Results from the GINIplus and LISAplus studies. Environ. Int..

[13] Kim S.-S., Park S.-A., Son K.-C. (2014). Improving Peer Relations of Elementary School Students through a School Gardening Program. HortTechnology.

[14] Oh Y.-A., Kim Y.-S., Park S.-A. (2019). Analysis of the Emotional Effects of Agricultural Experience Program Based on Social Emotional Learning Theory in Elementary School Students. J. Korean Soc. Rural. Plan..

[15] Oh Y.-A., Lee A.-Y., An K.J., Park S.-A. (2020). Horticultural therapy program for improving emotional well-being of elementary school students: An observational study. Integr. Med. Res..

[16] Park S.A., Cho M.K., Yoo M.H., Kim S.Y., Im E.A., Song J.E., Lee J.C., Jun I.G. (2016). Horticultural activity program for improving emotional intelligence, prosocial behavior, and scientific investigation abilities and attitudes in kindergarteners. HortTechnology.

[17] Kim K.-H., Park S.-A. (2018). Horticultural therapy program for middle-aged women’s depression, anxiety, and self-identify. Complement. Ther. Med..

[18] Lee A.-Y., Kim S.O., Gim G.M., Kim D.S., Park S.-A. (2019). Care Farming Program for Family Health: A Pilot Study with Mothers and Children. Int. J. Environ. Res. Public Health.

[19] Park S.-A., Son S.Y., Lee A.-Y., Park H.-G., Lee W.-L., Lee C.H. (2020). Metabolite Profiling Revealed That a Gardening Activity Program Improves Cognitive Ability Correlated with BDNF Levels and Serotonin Metabolism in the Elderly. Int. J. Environ. Res. Public Health.

[20] Han A.-R., Park S.-A., Ahn B.-E. (2018). Reduced stress and improved physical functional ability in elderly with mental health problems following a horticultural therapy program. Complement. Ther. Med..

[21] Lee S.-S., Park S.-A., Kwon O.-Y., Song J.-E., Son K.-C. (2012). Measuring Range of Motion and Muscle Activation of Flower Arrangement Tasks and Application for Improving Upper Limb Function. Korean J. Hortic. Sci. Technol..

[22] Park S.-A., Shoemaker C.A., Haub M.D. (2009). Physical and Psychological Health Conditions of Older Adults Classified as Gardeners or Nongardeners. HortScience.

[23] Park S.-A., Lee A.-Y., Kim J.-J., Lee K.-S., So J.-M., Son K.-C. (2014). Electromyographic Analysis of Upper and Lower Limb Muscles during Gardening Tasks. Korean J. Hortic. Sci. Technol..

[24] Son K., Song J., Um S., Lee J., Kwack H. (2004). Effects of visual recognition of green plants on the changes of eeg in patients with schizophrenia. Acta Hortic..

[25] Park S.-A., Song C., Choi J.-Y., Son K.-C., Miyazaki Y. (2016). Foliage Plants Cause Physiological and Psychological Relaxation as Evidenced by Measurements of Prefrontal Cortex Activity and Profile of Mood States. HortScience.

[26] Igarashi M., Aga M., Ikei H., Namekawa T., Miyazaki Y. (2015). Physiological and Psychological Effects on High School Students of Viewing Real and Artificial Pansies. Int. J. Environ. Res. Public Health.

[27] Oh Y.-A., Kim S.-O., Park S.-A. (2019). Real Foliage Plants as Visual Stimuli to Improve Concentration and Attention in Elementary Students. Int. J. Environ. Res. Public Health.

[28] Studente S., Seppala N., Sadowska N. (2016). Facilitating creative thinking in the classroom: Investigating the effects of plants and the colour green on visual and verbal creativity. Think. Ski. Creat..

[29] Chang C.-Y., Chen P.-K. (2005). Human Response to Window Views and Indoor Plants in the Workplace. HortScience.

[30] Hartig T., Evans G.W., Jamner L.D., Davis D.S., Gärling T. (2003). Tracking restoration in natural and urban field settings. J. Environ. Psychol..

[31] Kim S.-O., Jeong J.-E., Oh Y.-A., Kim H.-R., Park S.-A. (2021). Comparing Concentration Levels and Emotional States of Children Using Electroencephalography during Horticultural and Nonhorticultural Activities. HortScience.

[32] Tarkka I., Hallett M. (1990). Cortical topography of premotor and motor potentials preceding self-paced, voluntary movement of dominant and non-dominant hands. Electroencephalogr. Clin. Neurophysiol..

[33] Heckman M.A., Weil J., De Mejia E.G. (2010). Caffeine (1, 3, 7-trimethylxanthine) in foods: A comprehensive review on consumption, functionality, safety, and regulatory matters. J. Food Sci..

[34] Okolo C., Omurta A. (2018). Use of dry electroencephalogram and support vector for objective pain assessment. Biomed. Instrum. Technol..

[35] Klem G.H., Lüders H.O., Jasper H.H., Elger C. (1999). The ten-twenty electrode system of the International Federation. The International Federation of Clinical Neurophysiology. Electroencephalogr. Clin. Neurophysiol. Suppl..

[36] Flores L.P. (2002). Occipital lobe morphological anatomy: Anatomical and surgical aspects. Arq. Neuro-Psiquiatr..

[37] Osgood C.E. (1952). The nature and measurement of meaning. Psychol. Bull..

[38] McNair D.M., Heuchert J.P., Shilony E. (2003). Profile of Mood States Manual: Bibliography.

[39] Sowndhararajan K., Cho H., Yu B., Kim S. (2015). Effect of olfactory stimulation of isomeric aroma compounds, (+)-limonene and terpinolene on human electroencephalographic activity. Eur. J. Integr. Med..

[40] Marzbani H., Marateb H.R., Mansourian M. (2016). Methodological Note: Neurofeedback: A Comprehensive Review on System Design, Methodology and Clinical Applications. Basic Clin. Neurosci. J..

[41] Gruzelier J. (2009). A theory of alpha/theta neurofeedback, creative performance enhancement, long distance functional connectivity and psychological integration. Cogn. Process..

[42] Cahn B.R., Polich J. (2006). Meditation states and traits: EEG, ERP, and neuroimaging studies. Psychol. Bull..

[43] Lee Y.J., Kim H.G., Cheon E.J., Kim K., Choi J.H., Kim J.Y., Kim J.M., Koo B.H. (2019). The analysis of electroencephalography changes before and after a single neurofeedback alpha/theta training session in university students. Appl. Psychophys. Biofeedback.

[44] McGuigan F.J., Andreassi J.L. (1981). Psychophysiology—Human Behavior and Physiological Response. Am. J. Psychol..

[45] Nehmad O.L. (1998). The end in sight: A look at the occipital lobe. Clin. Eye Vis. Care.

[46] Nolte J. (1993). The Human Brain.

[47] Hutchison M. (1986). Megabrain: New Tools and Techniques for Brain Growth and Mind Expansion.

[48] Ryu H., Ko W., Kim J., Kim S., Kim M. (2013). Electroencephalography Activities Influenced by Classroom Smells of Male High School. Sci. Emot. Sensib..

[49] Elsadek M., Liu B., Lian Z. (2019). Green façades: Their contribution to stress recovery and well-being in high-density cities. Urban For. Urban Green..

[50] Hassan A., Chen Q., Liu L., Tao J., Li G., Jiang M., Li N., Lv B. (2019). Psychological and physiological effects of viewing a money plant by older adults. Brain Behav..

[51] Adams F.M., Osgood C.E. (1973). A Cross-Cultural Study of the Affective Meanings of Color. J. Cross-Cultural Psychol..

[52] Clarke T., Costall A. (2008). The emotional connotations of color: A qualitative investigation. Color Res. Appl..

[53] Wexner L.B. (1954). The degree to which colors (hues) are associated with mood-tones. J. Appl. Psychol..

[54] Lichtenfeld S., Elliot A.J., Maier M.A., Pekrun R. (2012). Fertile Green. Pers. Soc. Psychol. Bull..

[55] Li X., Zhang Z., Gu M.M., DongYue J., Jia W., YingMin L., HuiTang P. (2012). Effects of plantscape colors on psycho-physiological responses of university students. J. Food Agric. Environ..

[56] Elsadek M., Fujii E. (2014). People’s psycho-physiological responses to plantscape colors stimuli: A pilot study. Int. J. Psychol.Behav. Sci..

[57] Wilson E.O. (1984). Biophilia.

[58] Jang H.S., Kim J., Kim K.S., Pak C.H. (2014). Human brain activity and emotional responses to plant color stimuli. Color Res. Appl..

